# Fractional Excretion of Potassium Predicts Remission in Adult Nephrotic Syndrome

**DOI:** 10.34067/KID.0000001058

**Published:** 2026-03-26

**Authors:** Satoshi Minami

**Affiliations:** Department of Genetics, The University of Osaka Graduate School of Medicine, Suita, Osaka, Japan

**Keywords:** AKI, CKD, kidney tubule

Accurately predicting remission of proteinuria in adults with nephrotic syndrome remains a critical but unmet need in clinical nephrology. Achieving remission is a key prognostic factor for long-term kidney and cardiovascular outcomes. Patients who achieve remission show stabilization of kidney function, a lower risk of thromboembolic events, and improved overall survival. By contrast, those who fail to achieve remission experience persistent proteinuria and sustained hypoalbuminemia, often with recurrent relapses, ultimately leading to CKD and progression to ESKD.^1^ Despite its importance, reliable predictors of remission are still lacking. Kidney biopsy remains the gold standard for determining histologic subtype, yet it is invasive, not always feasible, and provides limited insight into the likelihood of treatment response. Thus, identifying noninvasive, reproducible, and physiologically meaningful biomarkers for remission prediction is of significant clinical value.

For decades, the fractional excretion of sodium (FENa) has been widely used to assess tubular function and intravascular volume status. However, its clinical utility has been increasingly questioned. In AKI, for example, multiple studies have shown that FENa is unreliable in patients exposed to diuretics and fails to consistently distinguish prerenal from intrinsic etiologies.^2,3^ This has prompted a search for alternative indices of tubular function. The fractional excretion of potassium (FEK) has emerged as a promising candidate.^4,5^ Because potassium secretion occurs almost exclusively in the distal nephron and is tightly regulated by aldosterone, sodium delivery, tubular flow, and epithelial sodium channels (ENaC) activity, FEK serves as a direct and integrative measure of distal tubular performance.^6^ Several reports in AKI suggest that FEK can outperform FENa in predicting persistent AKI, highlighting its capacity to capture maladaptive tubular physiology.^4^ Whether this concept applies to nephrotic syndrome warrants further study.

The retrospective cohort study featured in this issue^7^ explored whether FEK could serve as a prognostic marker in adult nephrotic syndrome. The investigators analyzed a large cohort encompassing diverse histologic subtypes such as minimal change disease (MCD), FSGS, and membranous nephropathy, evaluating the association between baseline FEK and subsequent remission of proteinuria. Patients with high FEK demonstrated significantly lower remission rates than those with low FEK. A cutoff value of 10% emerged as an optimal threshold, physiologically plausible—approximating the median FEK of about 8% in healthy adults—although external validation remains warranted. Subgroup analyses reinforced the robustness of these findings, showing that elevated FEK and lower remission rates were consistent across histologic subtypes. This cross-histologic robustness underscores the potential of FEK as a universal functional marker of tubular maladaptation, capturing a shared physiologic axis underlying diverse forms of nephrotic syndrome.

The usefulness of FEK as a predictor does not stem merely from reflecting proteinuria severity. Rather, FEK should be regarded as an integrative physiologic output representing the composite influence of nephron reserve, tubular adaptability, the degree of renin-angiotensin-aldosterone system (RAAS) activation, and proteinuria-induced tubular stress. A key to understanding FEK lies in the dynamics of sodium delivery to the distal nephron and the coupled Na^+^/K^+^ exchange that follows. In distal tubular segments, sodium is reabsorbed through ENaC, generating a lumen-negative potential difference that drives potassium secretion *via* renal outer medullary potassium (ROMK) channels. This tight coupling is precisely regulated by aldosterone, which enhances both ENaC and ROMK expression and activity, fine-tuning distal tubular Na^+^/K^+^ exchange. Consequently, FEK is not a static reflection of potassium excretion capacity but a dynamic parameter that encapsulates both sodium load delivered distally and aldosterone-dependent Na^+^/K^+^ exchange. In essence, fluctuations in FEK mirror the kidney's functional resilience—the coordinated adaptability of nephron architecture, tubular sodium handling, and hormonal responsiveness under stress.

In remission-prone forms—often represented by MCD—the glomerular and tubular structures remain largely intact, and nephron number is preserved. Consequently, proximal tubular sodium reabsorption remains highly efficient, reclaiming most filtered sodium even under strong RAAS activation, thereby minimizing distal sodium delivery and attenuating ENaC-mediated uptake and coupled ROMK-dependent secretion. Aldosterone responsiveness remains physiologically appropriate, ensuring finely tuned coordination between ENaC and ROMK activity. Together, these features prevent excessive potassium loss and maintain electrolyte homeostasis, leading to characteristically low FEK values that reflect an adaptive RAAS response and preserved tubular integrity. Accordingly, low FEK serves as a functional signature of preserved tubular integrity and strong remission potential.

By contrast, in remission-resistant forms, nephron loss, glomerular injury, and chronic interstitial inflammation lead to compensatory hyperfiltration and impaired proximal sodium reabsorption. Increased distal sodium delivery activates ENaC-mediated reabsorption and enhances ROMK-driven potassium secretion *via* the resulting lumen-negative potential. Compounding this, aldosterone sensitivity is frequently upregulated, arising from (*1*) chronic RAAS activation and sustained aldosterone exposure, (*2*) proinflammatory cytokine–mediated upregulation of mineralocorticoid receptor expression and activity, and (*3*) adaptive overexpression of ENaC and ROMK in response to increased distal sodium load. Together, these mechanisms drive excessive Na^+^/K^+^ exchange, resulting in maladaptive potassium wasting and elevated FEK. In this context, high FEK may indicate residual nephron overload and chronic maladaptive activation of the RAAS-ENaC-ROMK axis, accompanied by ongoing tubular stress, inflammation, and diminished reversibility. Although these mechanistic distinctions illustrate how FEK may arise from differing pathophysiologic contexts, this study indicates that its prognostic relevance extends across histologic categories—capturing a shared axis of tubular maladaptation underlying diverse nephrotic phenotypes. Collectively, these insights suggest that FEK reflects not only disease activity but also a broader tubular functional continuum that transcends histologic classification.

Proteinuria may further contribute to this maladaptive cycle *via* the plasminogen-plasmin-ENaC pathway, supported by experimental studies but not yet quantified clinically.^8^ Within the tubular lumen, filtered plasminogen is converted into active plasmin, which directly stimulates ENaC on the apical membrane of distal tubular cells. This promotes sodium reabsorption and coupled potassium secretion independently of aldosterone, elevating FEK. Importantly, this mechanism reveals a qualitative dimension of proteinuria beyond its quantity, showing how urinary proteins act as bioactive mediators reshaping tubular signaling and sustaining nephrotic physiology even without overt inflammation. In this setting, an elevated FEK indicates that proteinuria itself has become a pathogenic driver, fueling sodium retention, potassium wasting, and persistence of the nephrotic state.

This framework clarifies that it is not RAAS activation *per se*, but the physiologic context that determines FEK behavior. When nephron reserve and tubular adaptability are preserved—as in MCD-RAAS—activation acts as a physiologic adaptation, maintaining potassium conservation and yielding low FEK. By contrast, when nephron number is reduced and tubular integrity compromised—as in non-MCD—persistent RAAS activation becomes maladaptive, driving excessive ENaC activation, potassium wasting, and elevated FEK. In this light, FEK represents a dynamic, context-dependent physiologic signal of the RAAS–ENaC axis, capturing the balance between adaptive homeostasis and maladaptive overload.

Clinically, the implications are substantial. Although kidney biopsy defines histologic subtype, it offers limited insight into remission potential. By contrast, FEK provides a noninvasive, easily measurable, and physiologically grounded index that reflects the kidney's functional adaptability. Low FEK indicates preserved nephron reserve and a greater likelihood of remission, while high FEK signifies maladaptive overload and refractory disease. Interpretation should, however, account for potential confounders—including diuretics (especially potassium-sparing agents), RAAS blockers, acid–base status, dietary potassium, and urine flow conditions—that can modulate distal potassium handling independent of disease activity. Patients with elevated FEK might be candidates for hypothesis-generating trials testing whether modulation of maladaptive RAAS–ENaC signaling—using mineralocorticoid receptor antagonists or ENaC inhibitors—could improve outcomes, with careful monitoring for hyperkalemia. Although FEK was assessed only at baseline in this study, future prospective studies should evaluate longitudinal FEK changes during and after remission induction to determine whether it can serve as a real-time biomarker of treatment response or relapse risk.

Several limitations must be acknowledged. This was a retrospective analysis, and causality cannot be established. FEK was measured at baseline only; systematic longitudinal data after remission were unavailable. It remains unclear whether FEK decreases in parallel with remission or whether persistent elevation signals ongoing subclinical risk. Moreover, although mechanistic plausibility is strong, direct clinical evidence linking aldosterone or ENaC activation to reduced remission rates in adult nephrotic syndrome remains limited. Similarly, the hypothesis that residual nephron overload directly impairs remission has not yet been confirmed in prospective cohorts. Thus, the present findings should be interpreted as hypothesis-generating, laying the foundation for future mechanistic and interventional studies.

In conclusion, FEK emerges here as far more than a simple electrolyte ratio. It is a composite physiologic signal integrating nephron reserve, RAAS activity, and proteinuria-driven tubular stress. Low FEK signifies preserved tubular adaptability and a greater likelihood of remission, whereas high FEK reflects maladaptive overload and diminished reversibility. By capturing the physiologic architecture that determines treatment response, FEK provides a functional dimension that complements—and potentially surpasses—histologic assessment. With prospective validation, FEK could become a cornerstone biomarker for functional stratification, guiding individualized therapy and ultimately transforming the prognostic landscape of adult nephrotic syndrome. Nonetheless, these findings should be interpreted as hypothesis-generating, pending mechanistic and prospective clinical validation.
